# Vertical Graphene-Based Biosensor for Tumor Cell Dielectric Signature Evaluation

**DOI:** 10.3390/mi13101671

**Published:** 2022-10-04

**Authors:** Bianca Tincu, Tiberiu Burinaru, Ana-Maria Enciu, Petruta Preda, Eugen Chiriac, Catalin Marculescu, Marioara Avram, Andrei Avram

**Affiliations:** 1 National Institute for Research and Development in Microtechnologies—IMT Bucharest, 126A Erou Iancu Nicolae, 077190 Bucharest, Romania; 2 Faculty of Applied Chemistry and Material Science, University “Politehnica” of Bucharest, 313 Splaiul Independenței, 060042 Bucharest, Romania; 3 University of Agronomic Sciences and Veterinary Medicine, 59 Mărăști, 011464 Bucharest, Romania; 4 Biochemistry-Proteomics Department, Victor Babes National Institute of Pathology, 99–101 Splaiul Independenţei, 050096 Bucharest, Romania; 5 Cell Biology and Histology Department, Carol Davila University of Medicine and Pharmacy, 8 Eroii Sanitari, 050474 Bucharest, Romania

**Keywords:** electrochemical sensor, vertical graphene, electrochemical impedance spectroscopy, tumor cells

## Abstract

The selective and rapid detection of tumor cells is of critical consequence for the theragnostic field of tumorigenesis; conventional methods, such as histopathological diagnostic methods, often require a long analysis time, excessive analytical costs, complex operations, qualified personnel and deliver many false-positive results. We are considering a new approach of an electrochemical biosensor based on graphene, which is evidenced to be a revolutionary nanomaterial enabling the specific and selective capture of tumor cells. In this paper, we report a biosensor fabricated by growing vertically aligned graphene nanosheets on the conductive surface of interdigitated electrodes which is functionalized with anti-EpCAM antibodies. The dielectric signature of the three types of tumor cells is determined by correlating the values from the Nyquist and Bode diagram: charge transfer resistance, electrical double layer capacity, Debye length, characteristic relaxation times of mobile charges, diffusion/adsorption coefficients, and variation in the electrical permittivity complex and of the phase shift with frequency. These characteristics are strongly dependent on the type of membrane molecules and the electromagnetic resonance frequency. We were able to use the fabricated sensor to differentiate between three types of tumor cell lines, HT-29, SW403 and MCF-7, by dielectric signature. The proposed evaluation method showed the permittivity at 1 MHz to be 3.63 nF for SW403 cells, 4.97 nF for HT 29 cells and 6.9 nF for MCF-7 cells.

## 1. Introduction

The cancer statistic from the International Agency for Research on Cancer (IARC), GLOBOCAN 2020, shows that breast and colon cancers are more frequently reported. According to recent global cancer estimates, breast cancer (11.7%) has become the most commonly diagnosed cancer type in the world: about 2.3 million women were diagnosed with breast cancer in 2020, followed by lung cancer (11.4 %) and colorectal cancer (10%). In 2020, breast cancer caused about 685,000 deaths (6.9%) and colorectal cancer caused 940,000 (9.4%) [1,2]. For this reason, the early detection of cancer can be a key step in its efficacious treatment. In cancer research, biomarkers for the specific detection of tumor cells are of great importance. Circulating tumor cells (CTCs) have been found in the blood of patients with numerous types of cancer, such as breast, lung, pancreatic, liver and colon cancer. The detection and quantification of the type and the number of tumor cells enable the early diagnosis of cancer. The number of CTCs also has significant clinical importance [3,4]. Epithelial cellular adhesion molecule (EpCAM) is the most widely used tumor-associated antigen because it is overexpressed in many types of cancer, including breast cancer and colorectal cancer [5,6,7]. EpCAM is a biomarker used in cancer diagnosis [6]. Additionally, generally EpCAM-based diagnostic methods depend on the use of an anti-EpCAM antibody [7].

Numerous traditional methods for tumor cell detection are reported, including: (a) in vitro detection methods: (a1) biological characteristics: (i) positive selection: EPHESIA CTC-Chip, CellSearch® system, MagSweeper™, CTC-Chip and Herringbone-Chip, IsoFlux, Adnatest® system, Velcro-like device, GEDI microdevice, DEPArray®; (ii) negative selection: leukocytes depletion kits, RosetteSep method, CTC-iChip; (iii) selection-free: Epic Sciences, AccuCyte®-CyteFinder®; and (a2) physical characteristics: (i) ISET (isolation by size of epithelial tumor cells); (ii) ScreenCell systems: electrical features (surface charge); (b) in vivo detection methods: (b1) optical imaging technology: MRI (magnetic resonance imaging), PAT (photoacoustic tomography), OCT (optical coherence tomography), CT (computed tomography) and PET (positron emission tomography), in vivo flow cytometry (IVFC): fluorescence IVFC (IVFFC) and photoacoustic IVFC (IVPAFC); (b2) scaffold implants; (b3) intravenous indwelling needle device; (b4) microfluidic chip [8,9,10].

Tumor cell detection remains challenging since these type of cells are extremely rare and the number of accessible samples is limited. Therefore, there is a crucial requirement to develop an innovative point-of-care device that is user-friendly, efficient, low cost and has high sensitivity [11]. Electrochemical sensors for tumor cell evaluation, compared with traditional tissue biopsy, which is considered the “gold standard”, can be used for significantly advanced strategies including the analysis of rare cells or molecules in blood samples [12,13,14]. For tumor cell detection, electrochemical biosensors offer a remarkable solution for cancer monitoring as a result of their high sensitivity, good performance and rapid real-time quantitative information. Electrochemical impedance represents a particularly useful, sensitive, label-free method for sensitive and specific tumor cell detection [15,16]. Recently, electrochemical biosensors modified with graphene for signal amplification in electrochemical detection methods have been reported for the detection of tumor cells by using cell-targeting biomolecules (antibody [17,18], peptide [19,20,21], DNA aptamer [22,23,24], SARS-CoV-2 [25,26]) on the surfaces of conductive electrodes.

**Figure 1:**
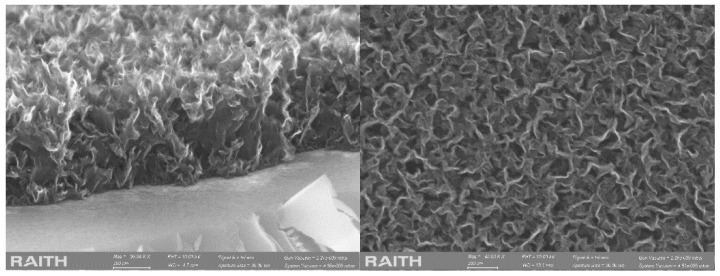
(**Left**) Cross-section image of VG grown on Si substrate; (**Right**) top view image of VG grown on Si substrate. The morphology of VG resembles vertical nanowalls with secondary nucleation sites on the vertical graphene sheets which results in a complex interconnected 3D morphology with secondary sheets growing from the sidewalls.

Graphene is a 2D material with carbon atoms arranged in a honeycomb structure and has been employed for sensor development due to its remarkable properties, such as: large surface area, excellent electrical conductivity, high thermal conductivity and chemical stability [27,28,29,30]. Graphene-based biosensors have been intensively investigated in enzymatic bio-sensing [31], DNA sensing, immunosensing [32] and cancer cell sensing [33,34]. Vertical graphene (VG) is a special form of graphene where the individual sheets grow perpendicular to the substrate. In contrast to pristine graphene, graphene oxide, reduced graphene oxide and similar 2D materials, vertical graphene (VG) sheets exhibit unique features such as non-agglomerated three-dimensional (3D) internetworked morphology, a large amount of open and ultrathin edges, and controllable structures [35], making them more appropriate for highly selective and sensitive biomarker detection in complex biological conditions. Figure 1 presents a cross-section and a top view of PECVD-grown VG, showing the distinctive morphology and structure that make its properties more interesting, such as readily accessible surface area, high electrochemical activity, and electric conductivity. Graphene-based electrochemical biosensors have been studied and discussed in many scientific papers over the past 20 years, but although vertically oriented graphene nanosheets have been known for at least 15 years, VG has been somehow sidelined for the more popular pristine graphene, graphene oxide and reduced graphene oxide. Until now, biosensors modified with VG have been reported for detecting various biomolecules including glucose, lactate, uric acid (UA), ascorbic acid (AA), DNA, DA, serotonin, and other small molecules [36,37,38,39].

Compared with pristine graphene, VG is directly grown on interdigitated electrodes, without the need for transfer from Cu foil to the working substrate. In our earlier study, we examined CVD single-layer graphene and vertical graphene as promising candidates for electrochemical biosensors, and we noticed the advantage of VG: more edges and defects which facilitate functionalization, and an increased number of attachment points for biological cells [40].

Electrochemical impedance spectroscopy (EIS) has been used to quantitatively analyze tumor cells by exhibiting frequency-dependent dielectric properties. As a representative study, Feng and co-workers fabricated an EIS cytosensor by using an epithelial cell adhesion molecule (EpCAM) aptamer-modified gold electrode that could specifically bind EpCAM-overexpressed MCF-7 cells. 

In this paper, we investigate the dielectric properties of tumor cells using an innovative EIS-based biosensor with interdigitated work microelectrodes modified with vertical graphene. Three different tumor cell lines have been tested and captured on gold electrodes covered with vertical graphene. Quantitative information was given by the changes in the electrochemical signal (EIS). The biosensor was tested on human breast cancer tumor cells (MCF-7) and two colorectal adenocarcinoma cell lines, SW-403 and HT-29. In comparison with previous work, our study shows a new approach of an electrochemical biosensor based on vertical graphene, enabling the specific and selective capture of three types of tumor cells. We were able to use the fabricated sensor to differentiate between three types of tumor cell lines—HT-29, SW403 and MCF-7—by dielectric signature.

## 2. Experimental Materials and Procedures

### 2.1. Materials and Instruments

Three cell lines were used to demonstrate the functionality of the vertical graphene electrochemical sensor to determine the dielectric signature: human colon adenocarcinoma cell line SW-403 (Cat. No. 87071008), HT-29 (Cat. No. 91072201), and breast cancer cells MCF-7 (HTB-22-ATCC) were purchased from the European Collection of Authenticated Cell Cultures (ECACC). Line SW-403 was cultured in RPMI-1640 culture medium (Bio Whittaker Lonza), line HT-29 was cultured in Mc-Coy’s 5A (Bio Whittaker Lonza), supplemented with 10% fetal bovine serum (FBS, Euroclone, South America) and 100 IU/mL penicillin + 100 µg/mL streptomycin (Lonza) (complete culture medium), and MCF-7 was routinely grown in RPMI with 10% SFB and 1% antibiotic–anti-mycotic solution, at 37 °C and 5% CO_2_.

Gold (III) chloride hydrate (HAuCl4∙3H2O, ≥ 99.9%), sodium citrate (C6H5Na3O7), cysteamine hydrochloride (CS), glutaraldehyde (GA, 50% in water), 3-aminopropyl-triethoxysilane solution (APTES) and phosphate-buffered saline (PBS) were purchased from Sigma-Aldrich, SUA, and anti-EpCAM antibodies (MA5-12436, Invitrogen, Waltham, Massachusetts, USA) and bovine serum albumin were also purchased (BSA, Sigma Aldrich, Auckland, New Zealand).

Scanning electron microscopy (SEM) was used for the morphological characterization of the working interdigitated electrodes modified with VG (vertical graphene). The surface morphology was evaluated with Nova NanoSEM 630 (FEI Company, Hillsboro, OR, USA).

Raman spectroscopy was used to evaluate vertical graphene growth on gold interdigitated electrodes. Raman spectra were obtained using the Witec Raman spectrometer (Alpha-SNOM 300 S, WiTec. GmbH, Kroppach, Germany) using a 532 nm diode-pumped solid-state laser with a maximum power of 145 mW. 

Electrochemical measurements were performed with Autolab PGSTAT204 with an FRA32M electrochemical impedance spectroscopy (EIS) module (METROHM AUTOLAB AG., Utrecht, The Netherlands) with AC amplitude 0.01 V.

### 2.2. Experimental Procedures

#### 2.2.1. Fabrication of Interdigitated Electrodes Modified with Vertical Graphene

**Figure 2:**
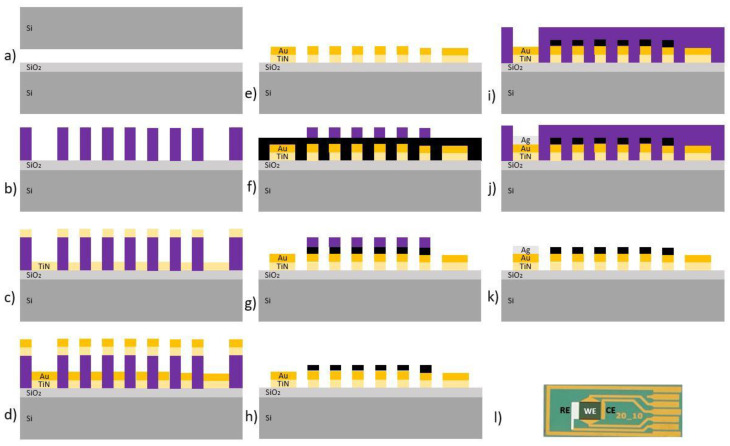
(**a**) Si/SiO_2_; (**b**) M1 photolithography; (**c**) TiN film deposition; (**d**) Au deposition; (**e**) Lift-off; (**f**) VG growth; (**g**) M2 photolithography; (**h**) VG etching; (**i**) photoresist removal; (**j**) M3 photolithography; (**k**) Ag deposition; (**l**) fabricated electrochemical sensor with Ag-coated reference electrode (RE), gold/VG working electrodes (WE), and gold counter electrode (CE).

The technological flow for the fabrication of the electrochemical sensor with interdigitated electrodes modified with vertical graphene is presented in Figure 2 and the main steps consist of:

(a) Substrate preparation: Si wafer cleaning by immersion in Piranha solution, followed by rinsing with deionized water and drying by centrifugation in nitrogen atmosphere; thermally grown silicon dioxide in wet atmosphere with a thickness of about 300 nm by thermal oxidation at 1000 °C. (b) M1 photolithography used for patterning the metallic layer configuration by the Lift-off process using 2 interdigitated working electrodes: counter electrode and reference electrode. (c) Fifty-nanometer titanium nitride layer (TiN) deposition by RF sputtering in order to fabricate a diffusion barrier. (d) Deposition of chromium (Cr) (10 nm) and gold (Au) (200 nm) by electron beam evaporation. (e) Lift-off process by dissolving the photoresist in acetone. (f) VG growth via plasma-enhanced chemical vapor deposition (PECVD—Nanofab 1000, Oxford Instruments, UK) at 700 °C, for 1h, in an Ar:CH_4_ atmosphere (partial pressure ratio 19:1). (g) M2 photolithography for the configuration of VG/interdigitated electrodes. (h) VG etching by O_2_ plasma in a reactive ion etching system (RIE—Etchlab 220, Sentech Instruments, Germany). (i) Photoresist removal and sample cleaning in acetone and isopropyl alcohol. (j) M3 photolithography for the deposition of the counter electrode. (k) A total of 200 nm silver (Ag) deposition by electron beam evaporation, and then Lift-off and cleaning. As a final step, a thin Al_2_O_3_ film was deposited on the electrical pathways from the electrodes to the contacting pads in order to insulate them during measurements. (l) Final device: electrochemical sensor.

#### 2.2.2. Functionalization of the Electrochemical Biosensor

Gold nanoparticles (AuNPs) were synthesized by a chemical method using 0.3 mMHAuCl_4_ as a metal precursor and 1% sodium citrate as a reducing agent. VG nanomaterial was modified with -NH_2_ group from the APTES structure by immersing the sensors in 10% APTES solution (prepared in deionized water) for 30 minutes at room temperature (25 °C). Then, sensors were washed and dried in an oven for 10 minutes at 120 °C in order to prevent the elution of APTES. Subsequently, the VG-based sensors modified with APTES were decorated with gold nanoparticles (about 30 nm) by electrostatic interaction through direct immersion in the AuNPs’ colloidal solution. The method was adapted from Nishida et al. (2007) [41] and Du et al. (2014) [42].

The immobilization of antibodies on the sensor surface was performed by conjugating AuNPs with 10 µg/mL of anti-EpCAM antibodies; the sensors were incubated with antibodies for 1 h. The immobilization of antibodies on AuNPs was mediated using 50 mM cysteamine hydrochloride (CS, prepared in PBS, pH 7.4) as a linker and 2.5% glutaraldehyde solution (GA, prepared in deionized water) was used to crosslink amino groups. VG/AuNP-based sensors were incubated with CS for 2 h and for 1 h with GA. After the immobilization of antibodies, 1% BSA solution was used for 30 minutes to minimize nonspecific adsorption. The method was adapted from Eissa and Zourob (2017) [43] and Layqah and Eissa (2019) [42]. All stages of the immobilization of antibodies were performed at room temperature in a humid atmosphere, and also the sensors were washed with PBS (pH 7.4).

#### 2.2.3. Cell Capture and Detection

**Figure 3:**
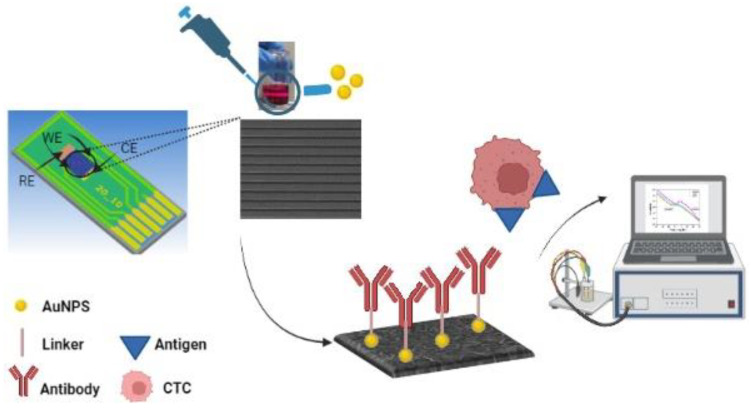
Electrochemical sensor: schematic presentation of the procedure of functionalizing the VG-based electrochemical biosensor and tumor cells’ capture and detection.

An aliquot of 20 µL of MCF-7, SW-403 and HT-29 cell suspension at a known concentration was transferred into the reaction chamber. The cells were allowed to adhere to the electrodes for 1 h before any measurements were performed, and then they were rinsed 3 times with PBS and measured in the presence of the electrolyte solution. The schematic representation of the tumor cells’ capture and detection can be seen in Figure 3.

## 3. Results

### 3.1. Morphological Characterization of Interdigitated Microelectrodes Modified with Vertical Graphene

**Figure 4:**
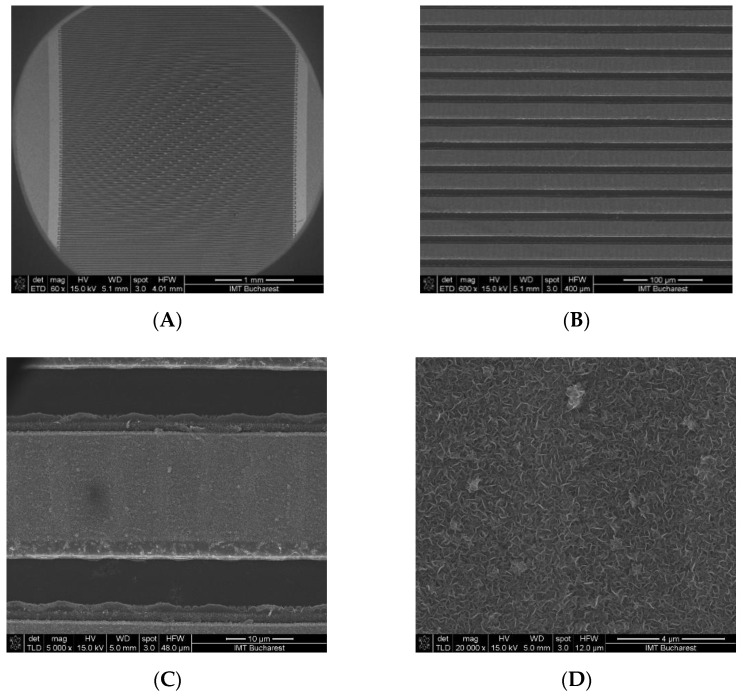

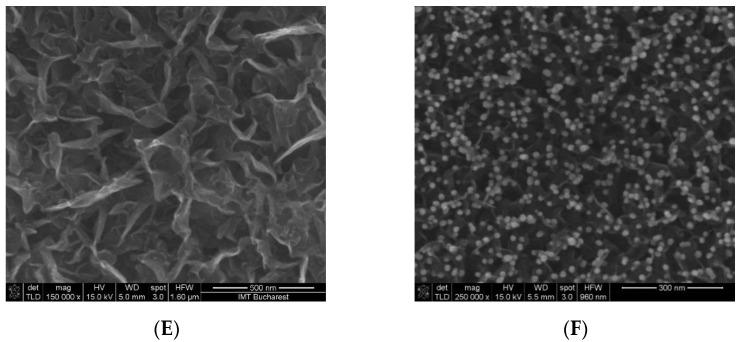
SEM micrographs of the vertical graphene working with interdigitated electrodes: (**A**) 60× magnification showing an overview image of the interdigitated electrodes; (**B**) 600× magnification showing a detailed image of the interdigitated working electrodes; (**C**) 5000× magnification showing a detailed image of a single electrode; (**D**) 20,000× magnification showing vertical graphene grown on the electrodes; (**E**) 150,000× magnification showing a detail of vertical graphene; (**F**) 250,000× magnification showing a detail of vertical graphene functionalized with gold nanoparticles.

To study the interdigitated working electrode structure and the morphology of VG growth on the gold electrodes, SEM micrographs of the interdigitated electrodes after the growth of VGs are shown in Figure 4A–E and the SEM images of the interdigitated electrodes after AuNP decoration of VG are shown in Figure 4F.

VG is vertically grown on the gold electrode and Figure 4C shows the presence of graphene and evidence of full coverage of the interdigitated electrodes. Figure 4E shows a representative SEM image of GNW morphology, suggesting the vertical growth of the two-dimensional carbon sheets on the electrode substrate. In Figure 4F it is observed that the AuNPs are immobilized and monodispersed on VG-based sensors, and that after the salinization process the morphology of VG was maintained.

### 3.2. Structural Characterization of Interdigitated Electrodes Covered with Vertical Graphene

**Figure 5:**
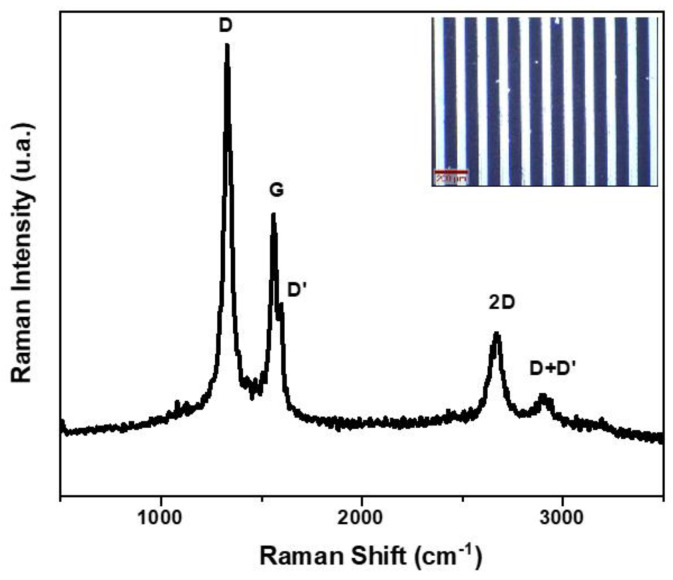
Raman spectrum of vertical graphene.

Raman spectroscopy was performed to determine the quality of VG growth on the gold electrode substrate. In Figure 5, a typical spectrum of VG is observed by the presence of specific bands: D at 1339 cm^−1^ and G at 1568 cm^−1^ (accompanied by D’ at 1608 cm^−1^) are very well outlined in the electronic noise, while the 2D band located at 2680 cm^−1^ is very well pronounced. The ratio I_D_/I_G_, representing the intensity ratio between the D and G bands, indicates the extent of overall defects present in the vertical graphene and can be attributed to the in-plane stretching of the carbon–carbon bonds, which can occur due to defects such as vacancies and edges [44]. In our case, the I_D_/I_G_ ratio of approximately 1.8 indicates a large number of defects which may be associated with the secondary or tertiary growth of additional graphene sheets nucleating on the already-growing sheets resulting in a branching growth, as correlated with the SEM images. The shape of the 2D band and the peak intensity ratio I_G_/I_2D_ of approximately 2.5 indicate that the thickness of the graphene sheets is formed by multiple layers, in the range of 7–10 atomic layers [45].

### 3.3. Tumor Cell Detection with Electrochemical Impedance Spectroscopy (EIS)

Tumor cell capture and detection using the developed sensor is based on the principle of antigen–antibody specific binding. Therefore, the fabricated VG-based electrochemical biosensor binds specifically to the EpCAM antigen via the anti-EpCAM antibody, which is the transmembrane glycoprotein found on tumor cells (Imrich et al., 2012) [46]. The modification of the surface of AuNPs with CS occurred through the thiol (-SH) group. GA was used for an amino group (-NH_2_) crosslinking from CS and an antibody chemical structure with the formation of the imine group (-CH = N-). Theoretically, functionalization with CS binds the antibodies to the surface with a random orientation. Therefore, the EpCAM/VG-based electrochemical biosensor specifically binds the MCF-7, HT-29 and SW-403 cancer cells immobilized on the VG electrodes via EpCAM presence in the cell membrane.

The purpose of analyzing EIS data is to determine the nature of the processes that take place at the electrode and their characteristic parameters. The transfer of electric charge is the basis of both components of the current: faradaic and non-faradaic. The faradaic component occurs in the case of electron transfer as a result of a reaction at the interface with the working electrode by overcoming a barrier called polarization resistance (Rp) in series with solution resistance (Rs). The non-faradaic current results from the charging of the capacitor formed by the electric double layer (Cdl). When charge transport takes place at the interface, the mass transfer of reactants and reaction products plays a role in determining ion transfer rates, which depend on the consumption of oxidants and the production of reductants near the surface of the electrodes. The transport of reactants and reaction products causes the occurrence of Warburg impedance (ZW), which in the Nyquist diagram is represented by a straight line with a slope of 45° at low frequencies. The activation of the barrier at any potential is represented by the polarization resistance (Rp), but at the point where the imaginary impedance is maximum, it becomes the resistance of the electric charge transfer (Rct). The kinetic effects occur at high frequencies and the mass transfer occurs at low frequencies. EIS can detect cell attachment, proliferation, and viability based on impedance and phase shift values. The EIS can provide a dielectric signature of the cell population, which is important for real-time cell monitoring.

The characterization of the cells using the developed device was performed by electrochemical impedance spectroscopy using an electrochemical impedance spectrometer PGSTAT204 with an FRA32 module (Metrohm Autolab AG, Utrecht, Netherlands). The modelling and analysis of the obtained data were performed in the first phase with the software of the spectrometer, followed by the processing of the initial data and the characterization of the biosensor in terms of electrochemical impedance. The impedance result models the conductance of the membrane in the area of the microelectrode matrix. When an electrical voltage is applied between the interdigitated working electrode and the auxiliary electrode of several tens of mV, on the digits of one of the electrodes, the anode, an oxidation reaction with the release of electrons occurs, and on the digits of the other, a reduction reaction with the capture of electrons takes place, and the solid–liquid interface behaves like a capacitor.

#### 3.3.1. Interpretation of the Nyquist Diagram Correlated with the Bode Diagram

The semicircle in the Nyquist diagram is due to the resistive coupling of the load transfer with the capacity of the electric double state at high frequencies. ZW and Rct depend on the concentration of the electroactive species. At low concentrations, the semicircle overlaps with diffusion, while at high concentrations the two processes are well separated. Rct can be associated with energy dissipation related to activation energy rather than an association with electron transfer. Therefore, the Nyquist diagram shows a competition between surface and volume diffusion, coupled with electric charge exchange processes, or poor adsorption coupled with surface diffusion. The maximum imaginary impedance (minimum capacitor capacity) at high frequencies is observed at the electron relaxation time τ_1_ = 1/ω_1_ = Rct·Cdl, and the minimum imaginary impedance (where the semicircle ends and the mass transfer begins) is observed at ion relaxation time τ_2_ = 1/ω_2_ = Rp·C, where C is the system capacity. C = Q/U, where Q is the total load stored in the capacitor, and U is the voltage applied to the capacitor terminals. Thus, a material with high electrical capacity stores a larger amount of electrical charge at the same potential difference.

Correlating the Nyquist and Bode diagrams, the relaxation times of the mobile charges can be determined. In the Bode diagram, the variation in the phase shift between current and voltage as a function of frequency is very important, because this curve gives information about the dielectric behavior of the molecules attached to the electrode. The inflexion point of the phase difference and the imaginary impedance is at the characteristic frequency at which the phase is −45°. If the phase difference tends towards zero for low frequencies, this indicates that the potential and current are in phase. If the phase difference tends towards zero for high frequencies, this indicates that the imaginary impedance is much lower than the real impedance. The phase difference (φ) is sensitive to the system parameters, thus providing an efficient way to compare the theoretical model with the experiment. The impedance modulus is sensitive to system parameters, but more so through asymptotic values at high frequencies, where it provides electrolyte resistance, and at low frequencies where it provides approximate resistance in DC.

It can be observed from the Nyquist diagram that when tumor cells were captured on the electrode, the electron transfer at the electrode–molecule interface was blocked, facilitating the quantification of tumor cells by the impedance modification, as evaluated by electrochemical impedance spectroscopy. The Faraday impedance of the equivalent electric circuit for charge-transfer control and diffusion control includes two parts: W, Warburg impedance, and the electrode polarization resistance, Rp. Rs, solution resistance, and Cdl, double-layer capacitance, are approximated as ideal circuit elements, and the Rp and W impedances are not ideal components; they have a certain relationship with the measuring frequency. Additionally, due to the vertical graphene-coated electrode surface, we replaced the ideal capacitor element (Cdl) with a constant-phase element (CPE), which can increase the quality of the electrical circuit fitting.

**Figure 6:**
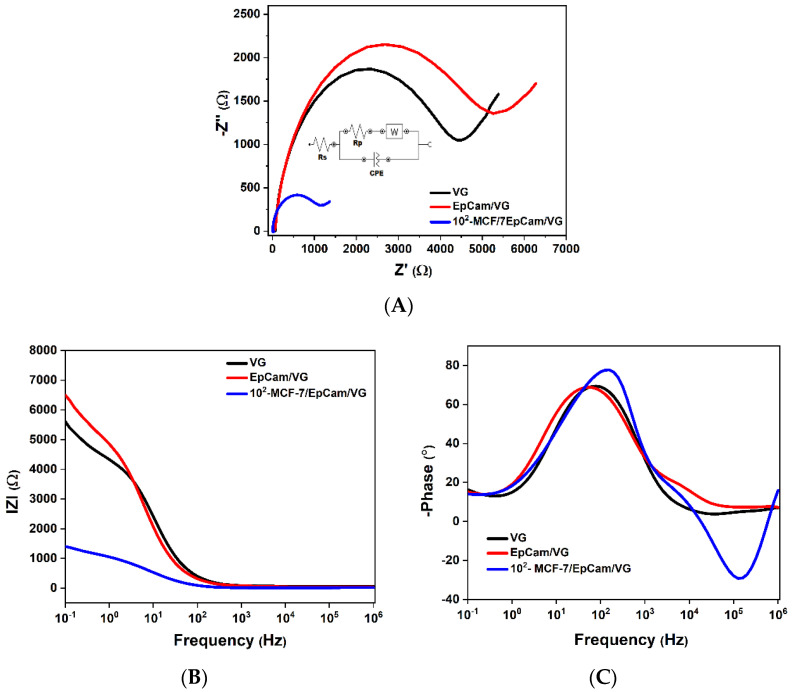
(**A**) Nyquist diagram; (**B**,**C**) Bode diagrams of VG: sensor blank, EpCam/VG and sensor after capturing 100 cell mL^−1^ concentration of MCF-7 cells from 0.1 to 10^6^ Hz in PBS solution containing 1 mM [Fe (CN)6]^3−/4−^ and 0.1 M KCl.

The impedance spectra (Nyquist plots) of the blank sensor (black curve), EpCAM/VG-based biosensor (red curve) and sensor after capturing MCF-7 cells (blue curve) are shown in Figure 6A. The impedance spectra are characterized by a semicircle portion at high frequencies, corresponding to the electron transfer-limiting process, and the radius of the semicircle is equal to the charge-transfer resistance (Rct). When the electrode surface is specifically functionalized, the charge-transfer process between the VG electrode and the electrolyte solution is disturbed, resulting in the charge-transfer resistance decreasing. The electrical charge of the cell membrane of MCF-7 is negative at physiological pH and, as shown in Figure 6A, the diameter of the semicircle decreases when the MCF-7 cells are immobilized by specifically attaching to the antibodies.

**Figure 7:**
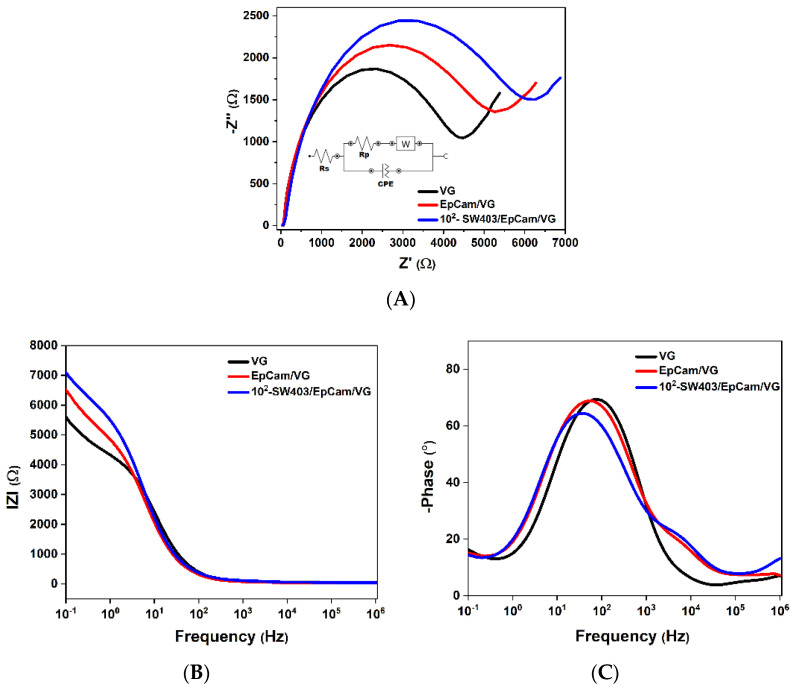
(**A**) Nyquist diagram; (**B**,**C**) Bode diagrams of VG: sensor blank, EpCam/VG-based sensor and sensor after capturing 100 cell mL^−1^ concentration of SW403 cells from 0.1 to 10^6^ Hz in PBS solution containing 1 mM [Fe (CN)6]^3−/4−^ and 0.1 M KCl.

The impedance spectra (Nyquist plots) of the blank sensor (black curve), EpCAM/VG-based biosensor (red curve) and sensor after capturing SW403 cells (blue curve) are shown in Figure 7A. The impedance spectra are characterized by a semicircle portion at high frequencies, corresponding to a high charge-transfer resistance. A fundamental property of the electric double layer is its electric charge, which can be altered by various metabolic transformations. For this reason, investigations of electric charge can provide considerable information on the equilibrium existing within the membrane and between the membrane and its environment, both in physiological and non-physiological conditions.

**Figure 8:**
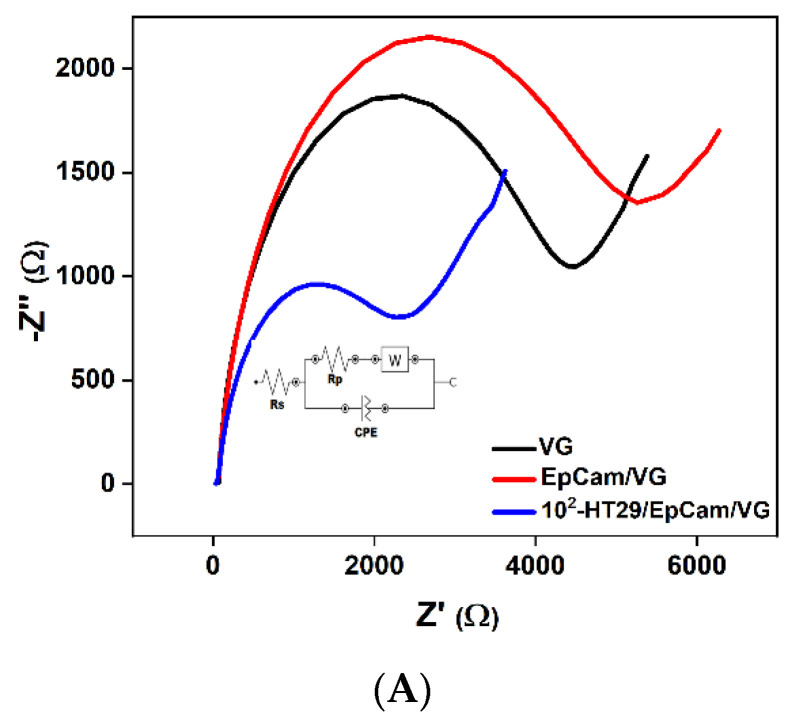

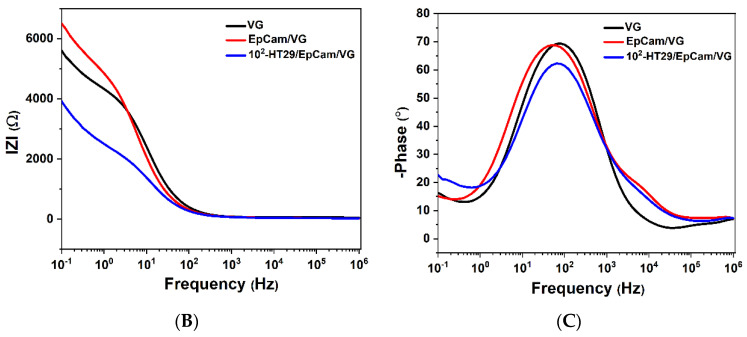
(**A**) Nyquist diagram; (**B**,**C**) Bode diagrams of VG: sensor blank, EpCam/VG-based sensor and sensor after capturing 100 cell mL^−1^ concentration of HT-29 cells from 0.1 to 10^6^ Hz in PBS solution containing 1 mM [Fe (CN)6]3^−^/4^−^ and 0.1 M KCl.

The impedance spectra (Nyquist plots) of the blank sensor (black curve), EpCAM/VG-based biosensor (red curve) and sensor after capturing HT-29 cells (blue curve) are shown in Figure 8A. When HT-29 cells are captured on the electrode surface, the charge-transfer process between the EpCam/VG electrode and the electrolyte solution is disturbed, resulting in the charge-transfer resistance decreasing.

#### 3.3.2. Interpretation of the Variation in Complex Dielectric Permittivity with Frequency

The result of the interaction of cells with the local electric field, by inducing the separation of charges with the formation of new dipoles, is the appearance of induced polarization. In biological systems, there are two important examples of induced polarization: ion migration on the surface of macromolecules, in other words, electric field-induced migration, and Maxwell–Wagner interfacial polarization on non-conductive surfaces. Biomaterials are composed of molecules that have large differences in permittivity and conductivity. When an electric field is applied to such a material, the mobility of charge carriers, such as ions, is considerably higher in some regions (e.g., aqueous phases) compared with other regions (such as lipid phases). This results in uneven load distribution on non-conductive surfaces. Such heterogeneous systems have strong frequency-dependent properties that are different from the properties of the phase constituents. In conclusion, the accumulation of electrical charges on the cell membrane can produce a Maxwell–Wagner-type interfacial relaxation process. This fact is observed experimentally with the help of the developed device by centering the dispersion—the dielectric loss curve—in the area of low frequencies. If the cell membrane contains electrically charged phospholipids, dielectric dispersion at high frequencies—but below 1 MHz—also become important. The presence of charged phospholipids attracts ion-charged populations of the opposite sign in the vicinity of the cell membrane. This dispersion is associated with both the movement of ions tangentially to the cell surface and the radial polarization of the ionic layers around it. The theory predicts that the relaxation time (τ) is proportional to the size of the cell and the mobility of nearby ions, while the maximum dielectric dispersion is proportional to the size and concentration of the cells.

The interaction of molecules with the electric field is characterized by complex permittivity. The real permittivity characterizes the material in terms of polarization, and the ideal permittivity characterizes the material in terms of dielectric loss. Dielectric permeability provides information about the opposite resistance to the electrical polarization of a dielectric (tumor cells being dielectric). Polarization can be achieved by the limited displacement of the bound electric charges or by the orientation of the existing dipoles in the material. The polarization can be: displacement polarization (electronic, ionic), orientation polarization (dipolar, electronic, ionic), resonant polarization and structural polarization. Of these, displacement polarization is excluded because it occurs at frequencies much higher than 1 MHz. Orientation polarization, or so-called relaxation, is typical of substances with polar molecules, which in the absence of the electric field are oriented chaotically under the influence of thermal energy. In the presence of the electric field, it is partially oriented. The energy dissipated in the unit of time in the material, under the influence of the electric field, constitutes losses in the dielectric, which are characterized by the imaginary permittivity. Molecular polarization arises due to the elongation of the chemical bond between atoms and the preferential orientation of molecules along the direction of the electric field. If the variations in polarization in time-varying electric fields are followed, experience shows that at low frequencies there is no comparison with polarization in the electrostatic field. It is necessary to distinguish between the induced polarization and the orientation of the permanent dipoles, which are two processes with totally different response times. The induced polarization of atoms and molecules occurs by deforming the electronic structure. Non-polar molecules behave identically from direct current to frequencies close to those of visible light, the polarization being proportional to the intensity of the electric field. Except for displacement polarizations, which are not accompanied by losses because the displacement of charged particles is elastic, all other polarization mechanisms occur with energy consumption. This energy is expended to overcome the resistance encountered in the orientation of the dipoles or to maintain the oscillations of electrons and ions. The polarization losses are proportional to the area of the electric induction cycle: the intensity of the applied electric field. Conduction losses occur in dielectrics that have high surface or volume conductivity.

The dielectric signature of cells represents the cellular response to the interaction with electromagnetic fields of different frequencies through interface phenomena that originate in the interaction of the electromagnetic field with membrane molecules. Phenomena at the interface of the electrode with biomolecules are enhanced by the functionalization of interdigitated gold electrodes with vertical graphene decorated with gold nanoparticles. This functionalization was chosen to increase the biosensing surface and conductivity at the electrode surface, knowing that graphene is a carbon nanomaterial with high electron mobility, and gold nanoparticles increase the concentration of electrons.

Different cell types can be distinguished because they have different phenotypes and different sets of molecules present on their membrane, thus expressing different genes and markers. This translates into similar but sufficiently different dielectric properties, allowing for differentiation between cell types.

According to the description of the cancer cell lines by the producer, SW-403 is a cell line exhibiting epithelial morphology isolated from the colon, and has no microvilli compared to HT-29 cells, which also have epithelial morphology but possess microvilli on their surface. Microvilli are specialized intestinal cells structures that protrude from the membrane, thus greatly increasing the absorbent surface of the cell and facilitating its role in the intestine. Because only HT-29 cells possess them, this means that they have a much larger cell membrane area. Additionally, microvilli have a very high concentration of specialized areas called lipid rafts and a wide array of proteins involved in nutrient absorption. This is why the cells are different when it comes to dielectric properties and EpCAM expression is irrelevant. EpCAM expression only comes into play when trying to capture them or characterize them from a mixture of cells. The MCF-7 cell line has a similar surface area to SW-403, but in turn its molecular membrane composition is widely different; it is rich in proteins, whereas SW-403 is rich in lipids.

## 4. Discussion

The data suggest that the electrical properties of breast cancer cells are different from those of the human colon adenocarcinoma cell lines. When the cells attach to the electrodes, the electric dipole moment is improved and the material becomes polarized. The ability of a material to interact with an electric field and become polarized is described in terms of its electric permittivity.

**Figure 9:**
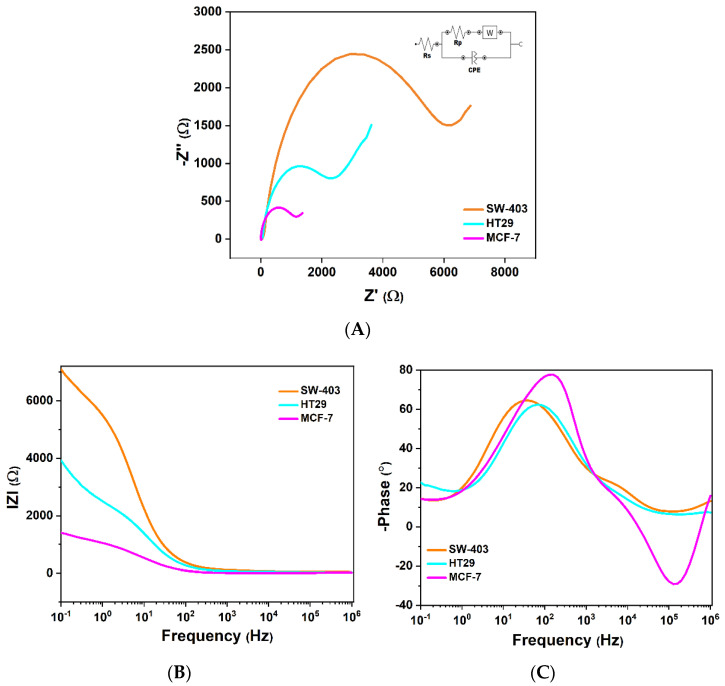
(**A**) Nyquist diagram; (**B**,**C**) Bode diagrams of the MCF-7, SW-403 and HT-29 cells from 0.1 to 10^6^ Hz in PBS solution containing 1 mM [Fe (CN)6]^3−/4−^ and 0.1 M KCl.

By fitting the measured impedance experimentally, the characteristic parameters from EIS are presented in Table 1. MCF-7 cells are large adherent cells, with a characteristic cell size measuring 20–25 µm. The influence of their size is observed in Figure 9A where MCF-7 cells have a lower charge transfer resistance, Rct = 551 Ω, and higher conductivity, Cdl = 18.8 µF, in comparison with HT-29 and SW-403. Both from the Nyquist diagram and the variation in the phase difference between current and voltage, we can observe that SW-403 cells compared to HT-29 and MCF-7 have: higher electrical charge transfer resistance, Rct = 3180 Ω, and lower permittivity and electrical conductivity. This can be explained by the fact that SW-403 cells adhere to one another in solution and form large cell conglomerates which impede the passage of current. Because of the microvilli in the cell membrane, the HT-29 cells have higher membrane capacity which results in the storage of a larger number of electrical charges, leading to higher conductivity and lower charge transfer resistance. At the level of microvilli, there is a very high concentration of lipid bonds, especially cholesterol–glycosphingolipids.

In this work, we exploit the cell differentiation capacity using the dielectric properties identified in Table 2. By calculating the relaxation time of the ions and electrons, we can determine the specific dielectric loss dependent on the cell type and morphology.

Double layer thickness (λ_D_) is calculated based on the dielectric polarization phenomenon at the electrode interface (Equation (6)).

The maximum imaginary impedance (Z″_max_) at high frequencies is observed at the relaxation time of the electrons (τ_1_), and the minimum imaginary impedance (where the semicircle ends and the mass transfer begins) is observed at the ion relaxation time (τ_2_). Dion is the ion diffusion coefficient at the DC limit and decreases with the concentration of cells.

An important method of investigating the dielectric signature of tumor cells is to characterize the interface between the cell membrane and the electrode. We study the effective thickness of the electrical double layer for MCF-7, SW-403 and HT-29 by solving the equations below:

(1) $$\tau_{1}=\frac{1}{2\pi \nu_{1}}$$

where *τ*_1_ is the relaxation time of the electrons and *ν*_1_ is the frequency for maximum imaginary impedance (Z″_max_);

(2) $$\tau_{2}=\frac{1}{2\pi \nu_{2}}\;$$

where τ_2_ is the relaxation time of the ions and ν_2_ is the frequency for minimum imaginary impedance (Z″_min_);

(3) $$\tau_{r}=\frac{1}{2\pi \nu_{r}}$$

where *τ_r_* is the relaxation time of electric charge and *ν_r_* is the resonance frequency;

(4) $$\delta =\frac{\tau_{2}}{\tau_{1}}$$

where *δ* is a dimensionless number describing dielectric loss;

(5) $$\tan\varphi_{max}=\frac{z^{\prime }}{z^{\prime \prime }}=\frac{\sqrt{\delta }}{2}$$

where tan *φ_max_* is the tangent of dielectric loss;

(6) $$\lambda_{D}=\frac{d}{\delta }\;$$

where *λ_D_* is the double layer thickness or Debye length and d is half the thickness of the electrode;

(7) $$D_{e}=\frac{\lambda_{D}^{2}}{\tau_{1}}$$

where *D_e_* is the electron diffusion constant;

(8) $$D_{i}=\frac{\lambda_{D}^{2}}{\tau_{2}}$$

where *D_i_* is the ion diffusion constant. The SW-403 tumor cell line adheres and forms large cell conglomerates observed by an increase in the area of the semicircle and a decrease in the value of the relaxation time, hence favoring high ionic conductivity and a high dielectric loss angle. The SW-403 tumor cell line has low *λ_D_* (14.5 nm) compared to HT-29, which has a much higher *λ_D_* of 57.8 nm and a lower capacitive reactance due to the microvilli. The double layer thickness of MCF-7 is around 18.3 nm.

Variations in the electrical permittivity of tumor cells result in permittivity variation. The polarization losses are proportional to the intensity of the applied electric field.

In biological samples, α dispersion occurs at low frequencies (below a few kHz), and β dispersion occurs at frequencies from tens of kHz to tens of MHz. The electrical properties of biological cells by their relative dielectric coefficients often approach extremely high values as the frequency decreases below 1 kHz. Cells are surrounded by a membrane, which separates the medium surrounding the cell from the cytoplasm. The principal mechanism responsible for the β dispersion is the accumulation of charges at the membrane level from extra and intracellular fluids (Maxwell–Wagner effect).

**Figure 10:**
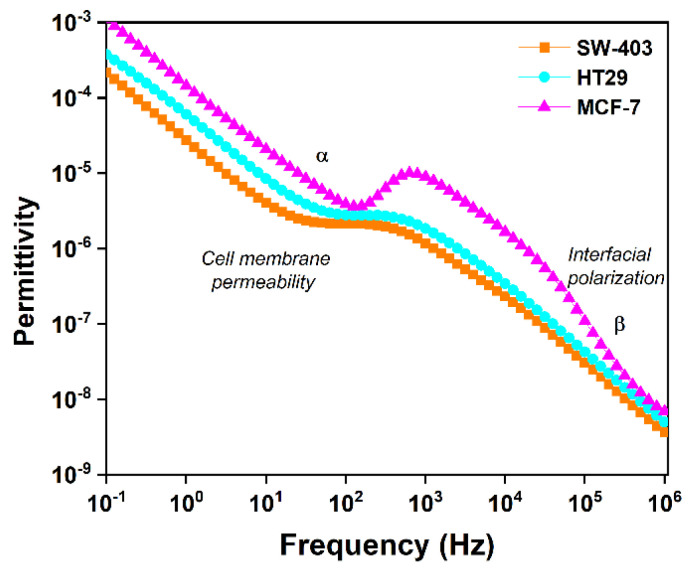
Mechanism of real permittivity dispersion for the different tumor cells: MCF-7, SW-403 and HT-29 cells.

Since the permittivity of the membrane decreases continuously with the frequency, the membrane capacitance shows a similar variation, as observed in Figure 10. Its value decreases from μF at 100 Hz to nF at 1 MHz.

The dielectric properties of tumor cells are frequency-dependent where α dispersion is due to ionic processes and β dispersion is due to charging up the membrane or orientation of permanent dipoles, as shown in Figure 10. In Figure 10, the fall in permittivity depending on the increase in the frequency is observed, which has a direct correlation with the integrity of the cell membrane and permittivity is dictated by the polarizability of the tumor cell type. The values for the real permittivity of the tumor cells at the frequencies 10^2^, 10^4^ and 10^6^ are extracted from Figure 10 to Table 3. Human breast cancer tumor cells (MCF-7) present a high permittivity in analogy with tumor cell lines from colorectal adenocarcinoma SW-403 and HT-29. Cell membrane permeability allows ion transport. β dispersion manifests differently due to the interfacial polarization of tumor cell membranes and polarization effects due to size and morphology differences for the tumor cells, which makes this frequency regime very relevant for the study of cells.

## 5. Conclusions

Electrochemical analysis has evidenced the functionality of this vertical graphene-based electrochemical biosensor, which detected the presence of tumor cells on the electrode surface at a low concentration: 100 cells mL^−1^. The data obtained by electrochemical impedance spectroscopy demonstrated that the HT-29 tumor cell line has a much larger cell membrane area and a lower capacitive reactance compared with the SW-403 cell line. The higher membrane capacity of HT-29 cells results in the storage of a larger number of electrical charges, which leads to higher conductivity and smaller charge transfer resistance. At the microvilli level, there is a very high concentration of lipid bonds, particularly cholesterol–glycosphingolipids, from which it can be deduced that HT-29 cells have a much higher concentration of lipid bonds compared with SW-403 cells. EIS measurements detect and confirm the fact that SW-403 tumor cells adhere and form large cell conglomerates. HT29 cell lines have a highly conductive surface and, therefore, upper dielectric losses are realized through conduction. MCF-7 cells being large adherent cells with a characteristic cell size measuring 20–25 µm have a lower charge transfer resistance and higher conductivity and permittivity in comparison with HT-29 and SW-403.

To highlight some of the limitations of the current work that will be addressed in future research, we mention the use of one breast cancer cell and two colon cancer cells, which in cases of cell signature evaluation might influence accuracy. Three tumor cells from the same origin (colon) could be a solution for accuracy improvement. Additionally, we intend to assess the influence of the cells’ concentrations on the results in our subsequent research.

## Figures and Tables

**Table 1:** Extracted impedance parameters found by fitting the experimentally measured impedance.

Sample	Rs (Ω)	Rp (Ω)	Rct (Ω)	Z″_max_ (Ω)	Cdl (μF)	Y_W_ (μS)
VG	53.8	2630	1474	1237	5.98	533
Functionalized VG	62.8	5020	2650	2123	6.10	685
SW-403	32.8	6070	3180	2740	6.38	722
HT-29	36.3	2410	1146	908	5.66	735
MCF-7	10.8	862	551	415	18.8	1800

**Table 2:** Calculated parameters found by fitting the experimentally measured impedance.

Sample	*τ*_1_ (s)	*τ*_2_ (s)	*τ*_r_ (s)	*δ*	tg max	d (m)	*λ_D_*	De (m^2^/s)	Di (m^2^/s)
SW-403	0.040	0.634	1 × 10^−6^	15.84	1.99	2.3 × 10^−7^	1.45 × 10^−8^	5.27 × 10^−15^	3.32 × 10^−16^
HT-29	0.025	0.100	8 × 10^−7^	3.98	1.00	2.3 × 10^−7^	5.78 × 10^−8^	1.32 × 10^−13^	3.32 × 10^−14^
MCF-7	0.032	0.400	1 × 10^−5^	12.58	1.77	2.3 × 10^−7^	1.83 × 10^−8^	1.05 × 10^−14^	8.34 × 10^−16^

**Table 3:** Real permittivity as a function of frequency for different tumor cells: MCF-7, SW-403 and HT-29.

Frequency	Permittivity (F)		
	SW-403	HT-29	MCF-7
10^2^	2.12 × 10^−6^	2.79 × 10^−6^	3.88 × 10^−6^
10^4^	2.37 × 10^−7^	4.42 × 10^−7^	1.66 × 10^−6^
10^6^	3.63 × 10^−9^	4.97 × 10^−9^	6.9 × 10^−9^

## Data Availability

All experimental data are available upon reasonable request from the corresponding authors via email.
